# *TNFSF14* and *CD44* are overexpressed in glioblastoma and associated with immunosuppressive microenvironment

**DOI:** 10.17305/bb.2025.11791

**Published:** 2025-02-13

**Authors:** Alja Zottel, Neja Šamec, Ivana Jovčevska

**Affiliations:** 1 Centre for Functional Genomics and Bio-Chips, Institute of Biochemistry and Molecular Genetics, Faculty of Medicine, University of Ljubljana, Ljubljana, Slovenia

**Keywords:** Glioblastoma, GBM, cluster of differentiation 44, *CD4*, tumor necrosis factor superfamily member 14, *TNFSF1*, immune-checkpoints, tumor microenvironment, TME

## Abstract

Glioblastoma (GBM) is one of the deadliest cancers, and the survival rate has remained low for decades. The aim of the study was the construction of the programmed death-ligand 1 (PD-L1) network, identification of its interactors and over-represented pathways, and analysis of the association between the identified genes and the immunosuppressive microenvironment of GBM. The PD-L1 network was constructed using Cytoscape and Search Tool for the Retrieval of Interacting Genes/Proteins (STRING). Over-representation analysis was performed on WebGestalt using Kyoto Encyclopedia of Genes and Genomes (KEGG), Protein ANalysis THrough Evolutionary Relationships (Panther), and Reactome Pathway Database (Reactome). Gene expression levels were examined *in silico* using three large datasets (The Cancer Genome Atlas (TCGA), Chinese Glioma Genome Atlas (CGGA), and Rembrandt), as well as with qPCR. The association between PD-L1 gene expression and immune cell infiltration was analyzed using the Tumor Immune Estimation Resource (TIMER 2.0) online tool. Cluster of differentiation 44 (*CD44*) and tumor necrosis factor superfamily member 14 (*TNFSF14*) were found to be significantly overexpressed in GBM compared to lower-grade glioma (LGG) and normal brain tissue. Their overexpression was associated with worse overall survival and demonstrated a strong ability to differentiate between GBM and reference brain tissue. Notably, *CD44* and *TNFSF14* were linked to the mesenchymal subtype of GBM and positively correlated with the presence of regulatory T cells, resting natural killer (NK) cells, and PD-L1 expression. Our findings highlight the overexpression of *CD44* and *TNFSF14* in GBM and their potential involvement in creating an immunosuppressive microenvironment. Unraveling the PD-L1 interaction network and its associated pathways offers the potential not only to identify novel biomarkers for GBM prognosis but also to pinpoint alternative therapeutic targets that could be more effective in overcoming the immunosuppressive hurdles inherent in GBM treatment.

## Introduction

With an incidence of 3.1 per 100,000 inhabitants [1], glioblastoma (GBM) is the most common primary brain tumor, accounting for 45.6% of brain tumors. The confirmed risk factors include exposure to high-dose ionizing radiation, age, race (white), and sex (male) [2, 3]. Additionally, certain genetic conditions, such as Li-Fraumeni syndrome and neurofibromatosis, contribute to GBM development [3]. GBM treatment has remained largely unchanged since 2005 and follows the Stupp protocol, which includes surgical tumor removal, radiotherapy, and chemotherapy with temozolomide [4]. Although some novel treatments, such as tumor-treating fields, lomustine, and regorafenib, have entered clinical practice [5–7], the prognosis for GBM remains exceptionally poor, with a median survival of just 15 months. This aggressive tumor is characterized by a high proliferation rate, core necrosis, microvascular proliferation, and tumor infiltration [8]. One of the key reasons therapy fails is the presence of the blood–brain barrier (BBB), which prevents most drugs from effectively penetrating the tumor [9]. Additionally, GBM is a highly heterogeneous tumor, consisting not only of differentiated cancer cells but also GBM cancer stem cells—highly malignant cells capable of forming new tumors, leading to recurrence [10, 11]. The tumor microenvironment (TME) of GBM is a complex network of various cell types, including differentiated cancer and stem cells, dendritic cells, pro-tumor macrophages, and other immune components, creating an immunosuppressive milieu that fosters tumor progression [12]. Notably, immune cells within the TME, such as regulatory T cells (Tregs) and natural killer (NK) cells, play significant roles in GBM malignancy. Tregs contribute to immunosuppression by downregulating cytokine IL-2 and facilitating the conversion of other T cells into Tregs [13, 14]. Conversely, NK cells are integral to the anti-tumor immune response, inducing apoptosis in target cells through granzyme B and perforin action [15]. However, glioma cells employ various strategies to diminish NK cell efficacy [16]. Tumor-associated macrophages (TAMs) and resident microglia, which can constitute up to 30% of the tumor mass, fluctuate between M1 (immunostimulatory) and M2 (immunosuppressive) phenotypes. A predominance of M2 macrophages correlates with a worse prognosis due to their role in promoting tumor angiogenesis and resistance to anti-VEGF therapies [17].

Although immunotherapy has shown promise in treating certain cancers, its efficacy in GBM has been largely disappointing, primarily due to the tumor’s highly immunosuppressive microenvironment. A key contributor to this immunosuppression is the PD-1/PD-L1 immune checkpoint pathway, which inhibits tumor cell apoptosis and promotes the conversion of effector T cells into Tregs. programmed death-ligand 1 (PD-L1, also known as CD274 and B7-H1) is a transmembrane protein encoded by the *PDCDL1* gene [18]. It consists of IgV and IgC extracellular domains, a hydrophobic transmembrane domain, and a cytoplasmic domain that functions as a signal transducer. *PD-L1* is expressed in various cell types, including tumor cells, antigen-presenting cells, B lymphocytes, and parenchymal cells, while its receptor, PD-1, is found on activated T cells. The interaction between PD-L1 and PD-1 facilitates immune evasion by suppressing T-cell activation and proliferation, reducing cytokine production, and inducing T-cell exhaustion [19]. Beyond its role in T-cell suppression, PD-L1 also contributes to epithelial–mesenchymal (ME) transition, chemoresistance, and the maintenance of stem cell properties [19]. Numerous clinical trials have evaluated anti-PD-L1 therapies for GBM, but results have been poor and inconsistent. Some studies have shown no significant benefit, while others have reported modest activity, particularly when PD-L1 inhibitors are combined with temozolomide and radiotherapy [20–23]. Although PD-L1 is frequently expressed in GBM, its expression levels vary widely, with a median of 2.77% positive cells, ranging from 0% to 86.6% [24]. So far, clinical trials targeting PD-L1 in GBM patients have yielded disappointing outcomes. In one trial, PD-L1 inhibition provided no benefit [20], while another study showed modest activity when combined with temozolomide and radiotherapy [23]. Specifically, the anti-PD-L1 drug Avelumab has failed to demonstrate any survival benefit for GBM patients, suggesting that PD-L1 may not be an optimal target for immunotherapy in this context [21, 22]. In response to these challenges, this study constructs and analyzes a PD-L1 interaction network, identifying overrepresented pathways and examining gene expression levels of pivotal genes. We employ in silico analyses using data from the Chinese Glioma Genome Atlas (CGGA), The Cancer Genome Atlas (TCGA), and Rembrandt databases, alongside experimental validation with human GBM, lower-grade glioma (LGG), and normal brain tissue samples. This comprehensive approach aims to uncover potential therapeutic targets and pathways that could enhance GBM treatment efficacy. Building on previous research, this study seeks to dissect the complexity of the PD-L1 interaction network within GBM. Our objective is to bridge the gap between the promising concept of immunotherapy and its practical application in improving GBM treatment outcomes, potentially paving the way for more effective, targeted therapies.

## Materials and methods

### Network construction and over-representation analysis

Two networks of PD-L1 (CD274) were constructed. The first network was generated using Cytoscape [25] by querying PubMed with the keywords “PD-L1” and “glioblastoma.” The confidence threshold was set to 0.7, and no more than 100 interactors were included. The second network was built using Search Tool for the Retrieval of Interacting Genes/Proteins (STRING) [26] with the search term “PD-L1,” also with a confidence threshold of 0.7, but limited to 50 interactors. Both networks were then merged in STRING to visualize their connections, without adding new neighbors. Over-representation analysis was performed using the WebGestalt online tool [27] with Kyoto Encyclopedia of Genes and Genomes (KEGG) [28], Protein ANalysis THrough Evolutionary Relationships (PANTHER) [29], and Reactome Pathway Database (Reactome) [30], using a genome-wide protein-coding reference set and an FDR cutoff of 0.05.

### In silico analysis

Several datasets were included in the *in silico* analysis: the CGGA mRNAseq 693 RSEM, TCGA; GBMLGG dataset obtained from Gliovis, and the Rembrandt dataset, also obtained from Gliovis. The CGGA database includes 693 glioma patients, comprising 249 with GBM, 188 with LGG, and 255 with grade 3 glioma. Patients were not stratified by sex, age, or mutation status. The TCGA dataset consists of 669 glioma patients of various grades, including 152 with GBM, 226 with LGG, and 244 with grade 3 glioma. Astrocytomas with no known grade were excluded from the study. As with CGGA, patients were not stratified by sex, age, or mutation status. The Rembrandt dataset includes 558 subjects, consisting of 219 patients with GBM, 100 with LGG, 85 with grade 3 glioma, and 28 non-tumor samples. Subjects with no known histology were excluded from the study.

For the CGGA and TCGA datasets, gene expression was analyzed in GBM, grade III glioma, and LGG. In the Rembrandt dataset, gene expression was assessed in GBM, grade III glioma, LGG, and non-tumor tissue. Differential gene expression analysis was performed in R (version 4.3.0) and RStudio (version 2023.03.1) using a one-way ANOVA test (function aov from the stats package). Survival analysis for GBM was conducted in R with subgroups defined by high and low gene expression levels, based on the optimal cutoff determined by surv_cutpoint (from the survminer package). Survival curves were visualized using ggsurvplot. Additionally, an ROC curve was generated using the Rembrandt dataset, comparing GBM patients with non-tumor subjects. This analysis was conducted in R with the pROC package.

**Table 1:** List of TaqMan probes used in the study

**Gene**	**Assay (ThermoFisher)**
*GAPDH*	Hs99999905_m1
*HPRT1*	Hs02800695_m1
*CASP4*	Hs01031951_m1
*CD276*	Hs00987207_m1
*CD44*	Hs01075864_m1
*RAB42*	Hs05288343_g1
*TNFRSF14*	Hs00187058_m1
*CD163*	Hs00174705_m1
*FKBP5*	Hs01561006_m1
*TNFSF14*	Hs00542477_m1
*CD40*	Hs01002915_g1
*ITGAM*	Hs00167304_m1
*PVR*	Hs00197846_m1
*TMEM205*	Hs00414441_m1
*TSTD1*	Hs00939899_g1
*CMTM6*	Hs00215083_m1
*HOXD13*	Hs00171253_m1

CD44: Cluster of differentiation 44; TNFSF14: Tumor necrosis factor superfamily member 14.

**Table 2:** List of 20 most over-represented pathways

**Description**	**Enrichment ratio**	**FDR**	**Database**
PD-1 signaling	95.1	0.0E+00	pathway_Reactome
Translocation of ZAP-70 to Immunological synapse	90.9	0.0E+00	pathway_Reactome
Phosphorylation of CD3 and TCR zeta chains	83.7	0.0E+00	pathway_Reactome
RUNX1 and FOXP3 control the development of regulatory T lymphocytes (Tregs)	57.6	4.4E−07	pathway_Reactome
Allograft rejection	54.5	0.0E+00	pathway_KEGG
Generation of second messenger molecules	52.3	0.0E+00	pathway_Reactome
GRB2 events in EGFR signaling	49.3	5.1E-04	pathway_Reactome
Graft vs host disease	44.9	0.0E+00	pathway_KEGG
Asthma	44.6	0.0E+00	pathway_KEGG
Costimulation by the CD28 family	44.4	0.0E+00	pathway_Reactome
SHC1 events in EGFR signaling	43.2	7.6E−04	pathway_Reactome
Interleukin-6 signaling	41.9	5.2E−05	pathway_Reactome
Type I diabetes mellitus	40.2	0.0E+00	pathway_KEGG
Intestinal immune network for IgA production	39.9	0.0E+00	pathway_KEGG
CD28 dependent Vav1 pathway	38.4	7.3E−05	pathway_Reactome
Autoimmune thyroid disease	36.9	0.0E+00	pathway_KEGG
TNFs bind their physiological receptors	35.7	9.7E−11	pathway_Reactome
CTLA4 inhibitory signaling	32.9	6.9E−07	pathway_Reactome
GAB1 signalosome	31.4	2.0E−03	pathway_Reactome
Inflammatory bowel disease (IBD)	30.1	0.0E+00	pathway_KEGG

Over-representation analysis was performed with Webgestalt online tool [27], using KEGG [28], Panther [29], and Reactome [30], using genome protein-coding reference set and cutoff FDR lower than 0.05. KEGG: Kyoto Encyclopedia of Genes and Genomes; PANTHER: Protein ANalysis THrough Evolutionary Relationships; Reactome: Reactome Pathway Database.

Protein expression of cluster of differentiation 44 (CD44) was determined using data from the Human Protein Atlas [31, 32]. Statistical analysis of protein expression was conducted using GraphPad Prism.

### Human tissue samples and gene expression analysis (qPCR)

The use of human tissue samples was approved by the National Medical Ethics Committee of the Republic of Slovenia (Approval Numbers: 92/06/12, 89/04/13, and 95/09/15). Written informed consent was obtained from patients prior to surgery. Reference samples were collected during autopsies in accordance with the legal regulations of the Republic of Slovenia. All samples used in this study are anonymized.

Expression levels of genes identified in silico were determined by qPCR in 29 GBM tissues, seven LGG tissues, and 12 reference tissues. RNA extraction had already been performed [33, 34]. Subsequently, 500 ng of RNA was treated with DNase I (Roche) under the following conditions: incubation for 15 min at 30 ^∘^C, followed by 10 min at 75 ^∘^C. The RNA was then transcribed into cDNA using the High-Capacity cDNA Reverse Transcription Kit (Thermo Fisher), with the addition of an RNAse inhibitor (1 µL/reaction; cat. no. N8080119, Thermo Fisher). The reaction conditions were as follows: 10 min at 25 ^∘^C, 120 min at 37 ^∘^C, and 5 min at 85 ^∘^C. For qPCR, the reaction mixture consisted of 0.25 µL of TaqMan assay, 2.5 µL of TaqMan mastermix, 2 µL of H_2_O, and 0.25 µL of cDNA. The cycling conditions were: initial denaturation at 95 ^∘^C for 20 s, followed by 45 cycles of 95 ^∘^C for 1 s and 60 ^∘^C for 20 s, with a final hold at 4 ^∘^C. The experiment was performed in three technical replicates. *GAPDH* and *HPRT1* were used as reference genes. The results were analyzed as previously described [35]. A list of TaqMan probes is provided in Table 1.

**Figure 1.**
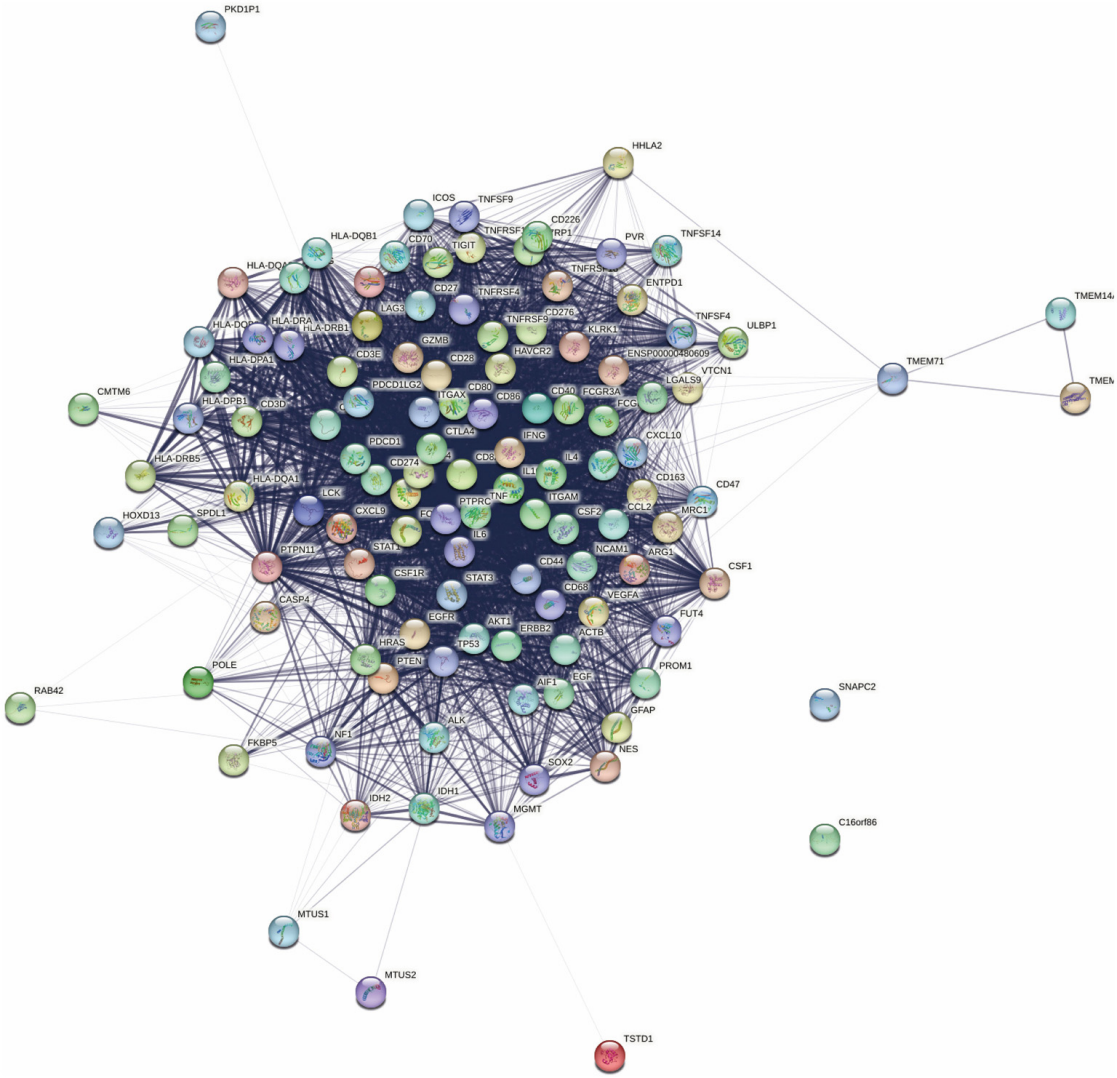
**STRING network of PD-L1 associated proteins.** It is a combination of two networks, performed with (1) Cytoscape and (2) String analysis analysis. The first network was created using Cytoscape and Pubmed query with keywords “PD-L1” and “glioblastoma”. The second network was constructed by String with the search term “PD-L1”. Confidence was set to 0.7 and no more than 50 interactors were included. Both networks were then combined in String to show their connection, without adding new neighbors. PD-L1: Programmed death-ligand 1; STRING: Search Tool for the Retrieval of Interacting Genes/Proteins.

Expression levels of genes identified in silico were determined by qPCR in 29 GBM tissues, seven LGG tissues and 12 reference tissues. The RNA extraction has been already performed [33, 34]. Afterwards, 500 ng of RNA was treated with DNAse l (Roche) with the following settings: incubation for 15 min at 30 ^∘^C and 10 min at 75 ^∘^C. Next, RNA was transcribed to cDNA with High-Capacity cDNA Reverse Transcription Kit (Thermo Fisher) and RNAse inhibitor (1 µL/reaction; cat. n. N8080119, Thermofisher) was added. The following settings were used: incubation for 10 min at 25 ^∘^C, 120 min at 37 ^∘^C and 5 min at 85 ^∘^C. The reaction mixture for qPCR was composed of 0.25 µL of TaqMan assay, 2.5 µL of TaqMan mastermix, 2 µL of H_2_O and 0.25 µL of cDNA. The settings were as follows: 20 s at 95 ^∘^C; 45 cycles of 1 s 95 ^∘^C, 20 s 60 ^∘^C; hold 4 ^∘^C. The experiment was performed in three technical replicates. The reference genes were *GAPDH* and *HPRT1*. The results were analyzed as described before [35]. The list of TaqMan probes is given in Table 1.

### Association of gene expression, immune cells infiltration, correlation with immune genes and expression across different tumors

The association between gene expression and immune cell infiltration was analyzed using the TIMER 2.0 online tool [36]. Only results from CIBERSORT, a method for characterizing cell composition, were included. Correlation analysis with immune-related genes was conducted in R (version 4.3.0) and RStudio (version 2023.03.1) using the stats package and the Spearman statistical test. Gene lists were obtained from the TISIDB online database [37], while gene expression data across different tumor types were retrieved from the GEPIA online database [38].

## Results

### Immune processes were identified as the most over-represented in the network and gene set enrichment analysis

In the first step, we constructed a PD-L1 network using Cytoscape and STRING (Figure 1). Pathway analysis (Table 2) revealed that the associated genes are primarily involved in various immune processes. The 20 most overrepresented pathways are listed in Table 2, with the top five being: (1) PD-1 signaling, (2) Translocation of ZAP-70 to the immunological synapse, (3) Phosphorylation of CD3 and TCR zeta chains, (4) RUNX1- and FOXP3-mediated regulation of regulatory T lymphocyte (Treg) development, and (5) Allograft rejection.

**Figure 2.**
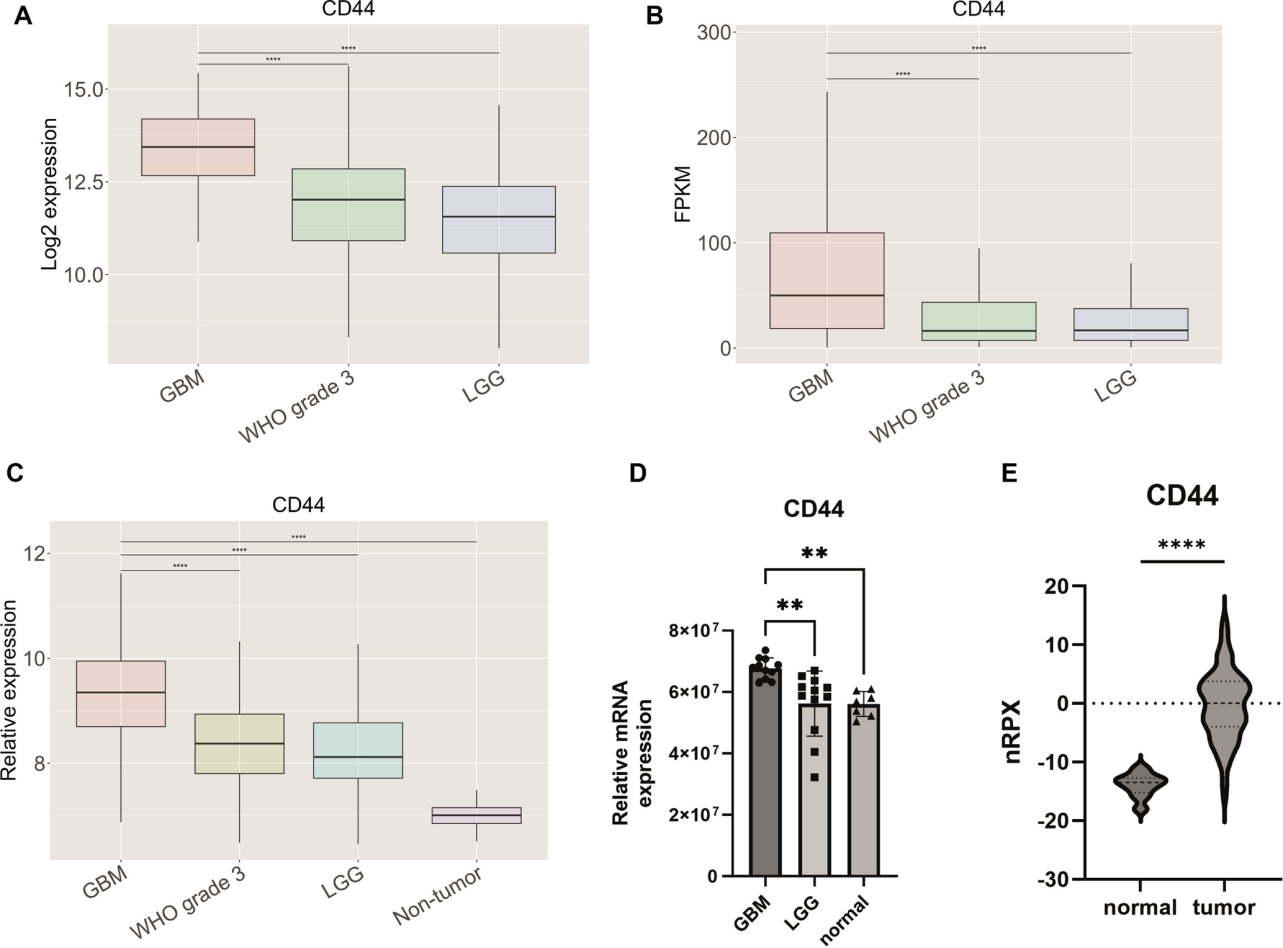
**Gene expression of *CD44*.** Gene expression was determined across three different datasets, (A) TCGA, (B) CGGA, and (C) Rembrandt. Gene expression levels were (D) experimentally validated by qPCR in human tissue samples. (E) Protein expression of CD44 is presented; results were obtained from the Human Protein Atlas [31, 32]. Results are presented as mean +/− SD. *P < 0.05, **P < 0.01, ***P < 0.001, ***P < 0.0001. CD44: Cluster of differentiation 44; TCGA: The Cancer Genome Atlas; CGGA: Chinese Glioma Genome Atlas.

**Figure 3.**
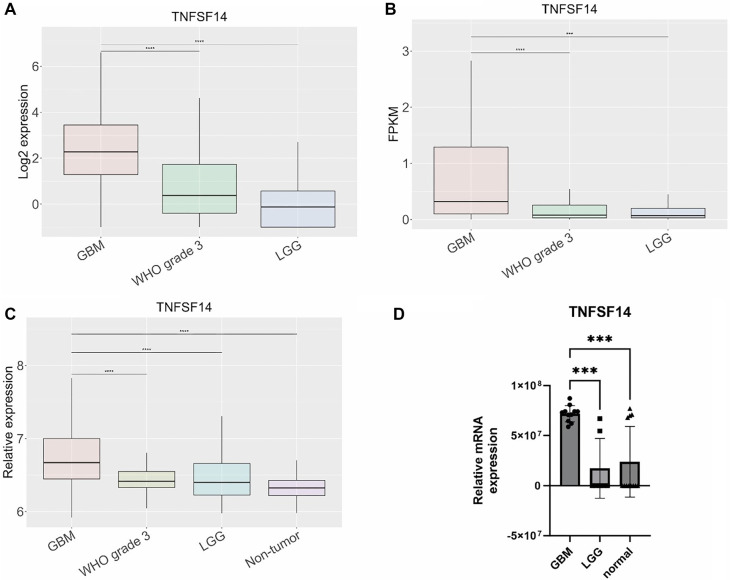
**Gene expression of *TNFSF14*.** Gene expression was determined across three different datasets, (A) TCGA, (B) CGGA, and (C) Rembrandt. Gene expression levels were (D) experimentally validated by qPCR in human tissue samples. Results are presented as mean +/− SD. *P < 0.05, **P < 0.01, ***P < 0.001, ***P < 0.0001. TNFSF14: Tumor necrosis factor superfamily member 14; TCGA: The Cancer Genome Atlas; CGGA: Chinese Glioma Genome Atlas.

**Figure 4.**
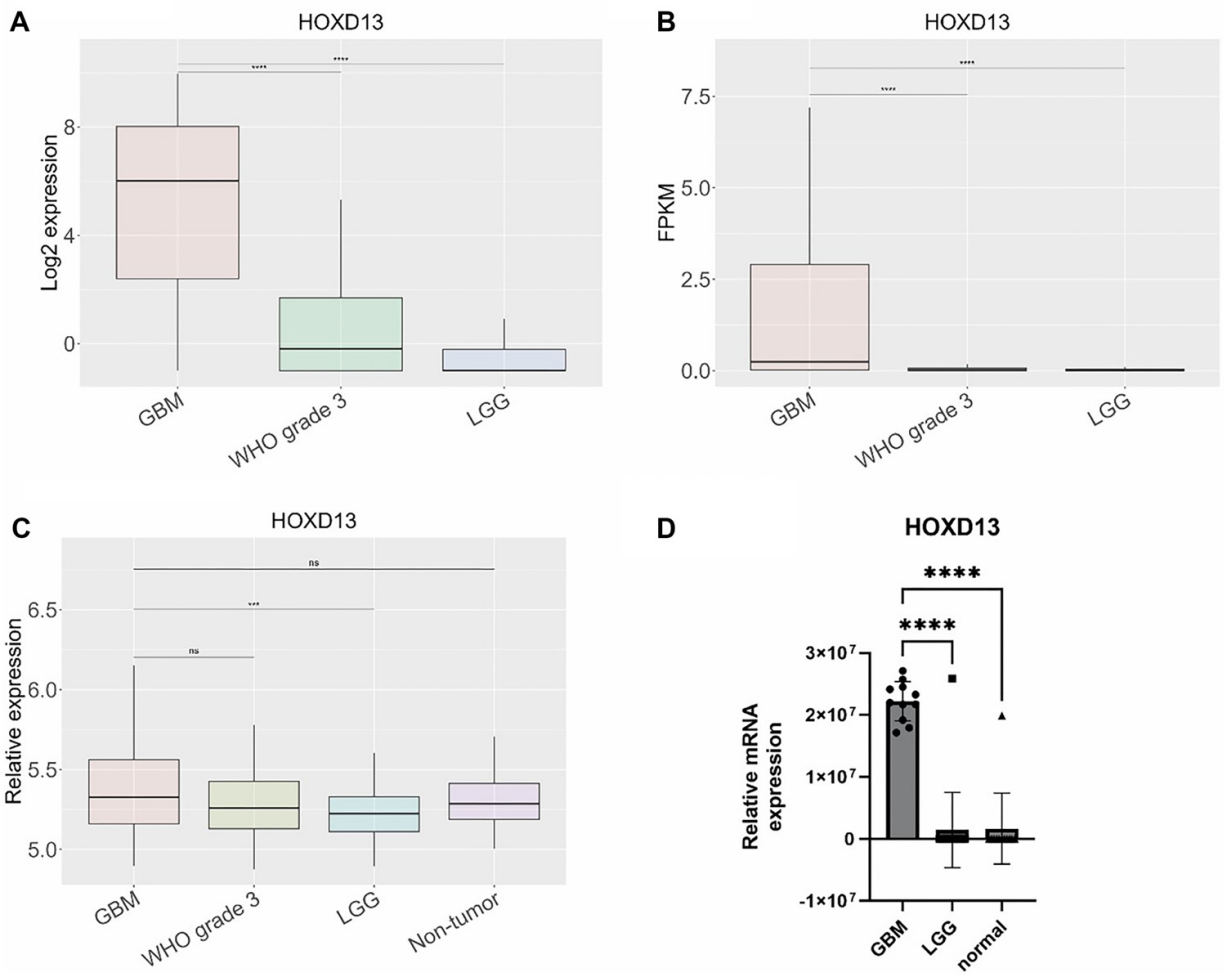
**Gene expression of *HOXD13*.** Gene expression was determined across three different datasets, (A) TCGA, (B) CGGA, and (C) Rembrandt. Gene expression levels were (D) experimentally validated by qPCR in human tissue samples. Results are presented as mean +/− SD. *P < 0.05, **P < 0.01, ***P < 0.001, ***P < 0.0001. TCGA: The Cancer Genome Atlas; CGGA: Chinese Glioma Genome Atlas.

### In silico selection of a set of genes overexpressed in GBM and correlated to worse overall survival

In the next step, we analyzed gene expression and its correlation with survival. Three datasets were included: CGGA, TCGA, and Rembrandt. The genes selected for analysis were identified through STRING and Cytoscape analysis, as described in Section 3.1.

From the results, we selected genes for further investigation based on two criteria.

**Figure 5.**
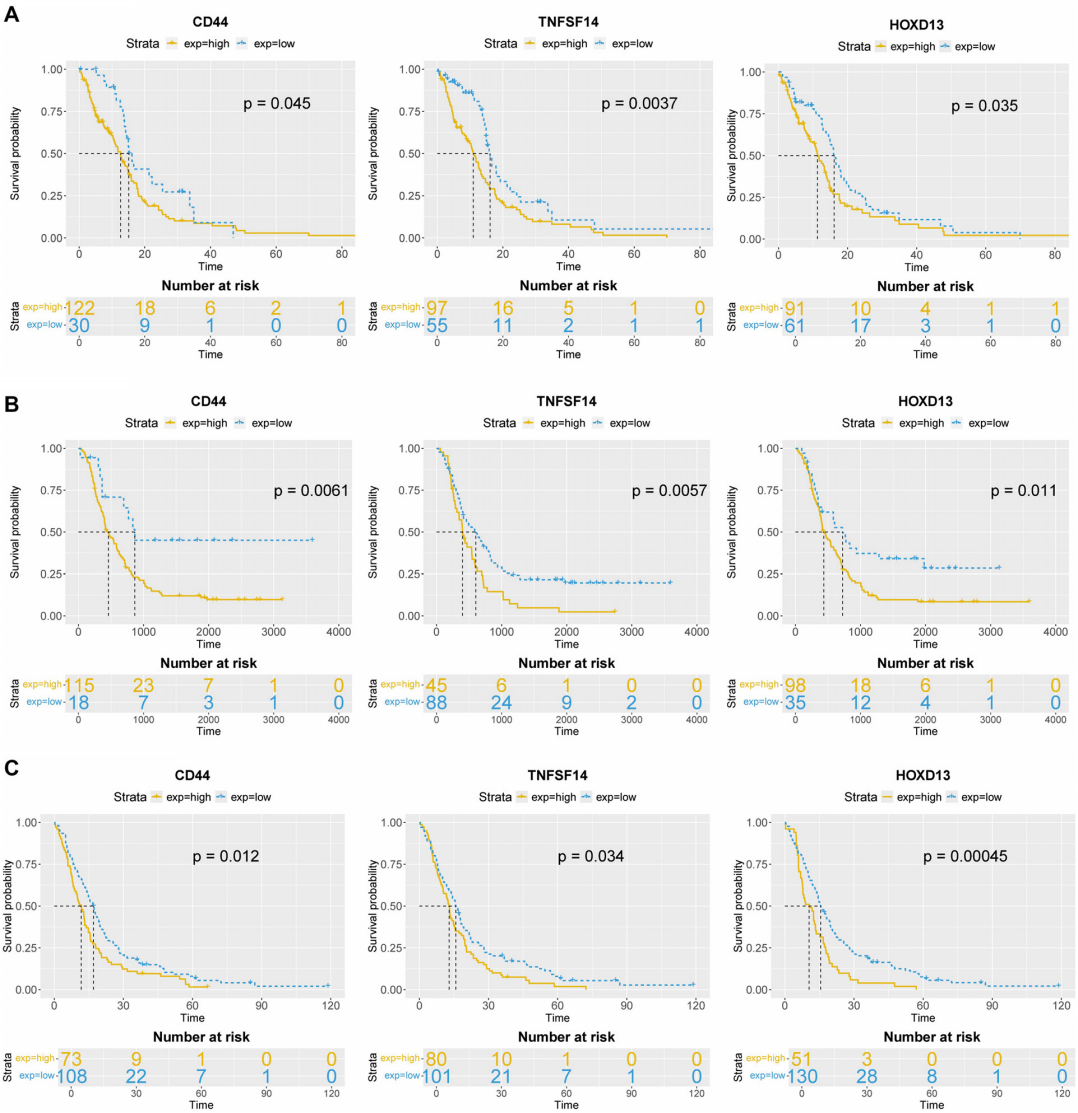
**Survival analysis of *CD44*, *TNFSF14,* and *HOXD13*.** Survival of CD44, TNFSF14 and HOXD13 was determined across (A) TCGA, (B) CGGA, and (C) Rembrandt datasets. CD44: Cluster of differentiation 44; TNFSF14: Tumor necrosis factor superfamily member 14; TCGA: The Cancer Genome Atlas; CGGA: Chinese Glioma Genome Atlas.

**Table 3:** Survival of GBM patients

	**TCGA**		**CGGA**		**Rembrandt**	
	**High**	**Low**	**High**	**Low**	**High**	**Low**
CD44	12.6	15.1	15.1 (459)	28.4 (866)	11.7	17
TNFSF14	11	16.1	13.2 (401)	19.8 (603)	12.9	15.8
HOXD13	11.3	16.1	14.4 (438)	23.8 (723)	11.5	15.6

The table presents median survival (months) in of GBM patients across three datasets (TCGA, CGGA, and Rembrandt) and divided into two groups (high and low gene expression). For CGGA, the median survival is presented in months and in brackets in days. GBM: Glioblastoma; CD44: Cluster of differentiation 44; TNFSF14: Tumor necrosis factor superfamily member 14; TCGA: The Cancer Genome Atlas; CGGA: Chinese Glioma Genome Atlas.

**Figure 6.**
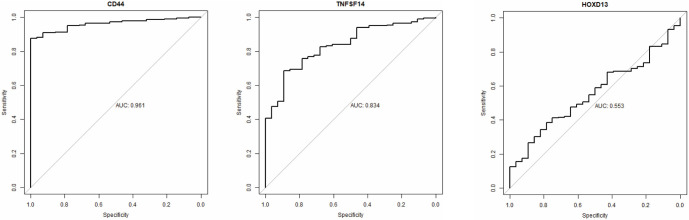
**ROC curve of *CD44*, *TNFSF14,* and *HOXD13*.** The AUC for *CD44* is 0.961, for *TNFSF14* 0.834 and for *HOXD13*, 0.553. CD44: Cluster of differentiation 44; TNFSF14: Tumor necrosis factor superfamily member 14.

**Figure 7.**
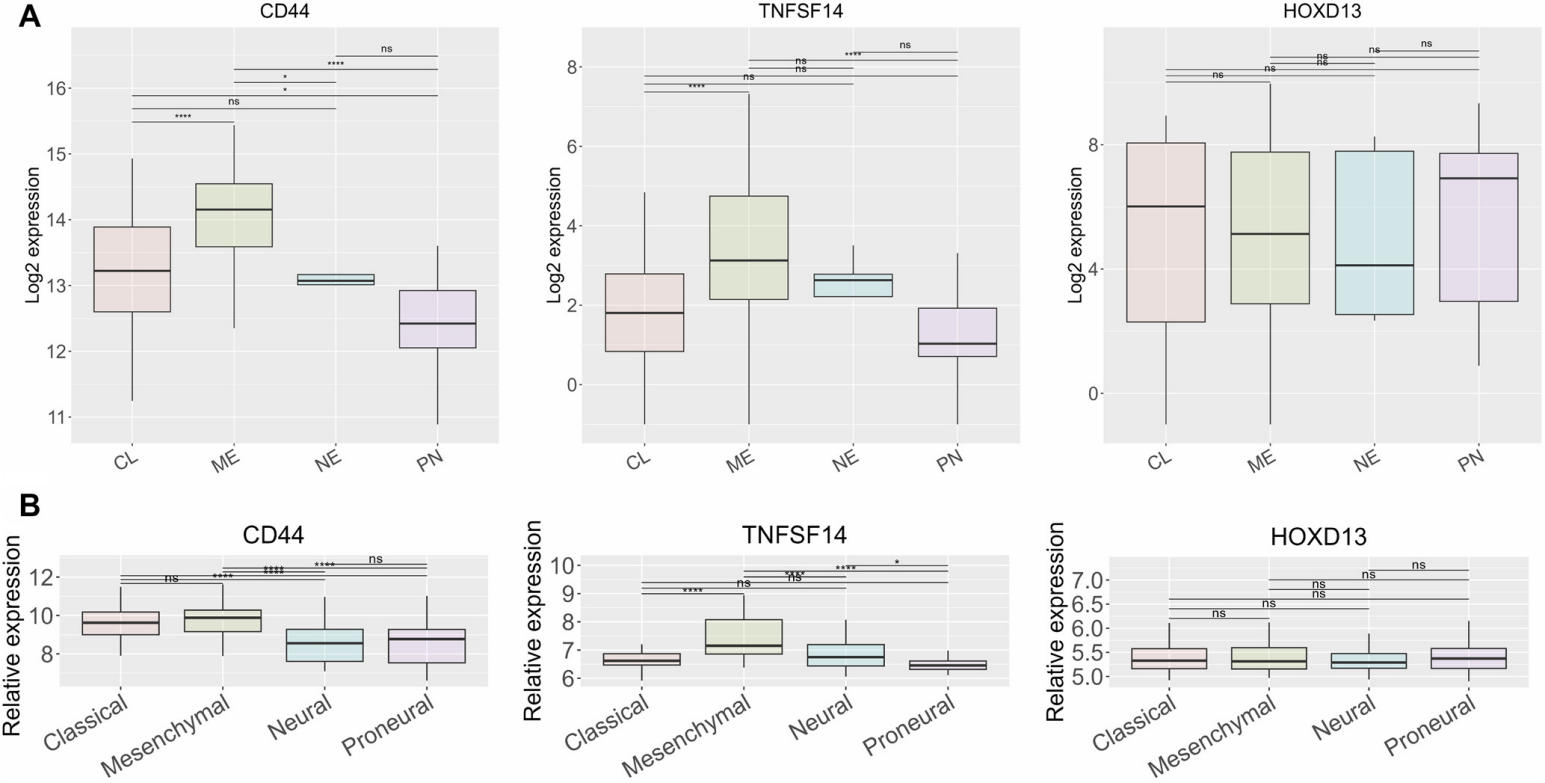
**Gene expression levels of *CD44*, *TNFSF14,* and *HOXD13* across different GBM subtypes.** Two datasets were included in the study, (A) TCGA and (B) Rembrandt. Four different subtypes were included, classical, mesenchymal, neural, and proneural. Results are presented as mean +/− SD. *P < 0.05, **P < 0.01, ***P < 0.001, ***P < 0.0001. CD44: Cluster of differentiation 44; TNFSF14: Tumor necrosis factor superfamily member 14; GBM: Glioblastoma.

They were overexpressed in GBM compared to WHO grade 3 glioma, LGG, and normal brain tissues (for Rembrandt only) in at least two datasets. Their higher expression was associated with worse overall survival in GBM patients across at least two datasets. The genes that met these criteria were *CASP4, CD40, CD44, CD163, CD276, CMTM6, FKBP5, HOXD13, ITGAM, PVR, RAB42,* tumor necrosis factor superfamily member 14 (*TNFSF14), TNFRSF14, TMEM205*, and *TSTD1*.

### *CD44*, *TNFSF14*, and *HOXD13* are overexpressed in GBM tissue samples and linked to worse overall survival

Among the 15 genes analyzed for expression levels using qPCR (Figures 2–4, Figure S1), only three—*CD44*, *TNFSF14*, and *HOXD13*—were found to be overexpressed in GBM compared to both LGG and normal brain tissue, as shown in Figures 2D, 3D, and 4D. *CD44* and *TNFSF14* were significantly overexpressed in GBM compared to WHO Grade 3 glioma (confirmed by TCGA, CGGA, and Rembrandt), as well as LGG and normal brain tissue (confirmed by Rembrandt and qPCR analysis). In addition to gene expression, CD44 was also found to be expressed at the protein level in GBM patients compared to controls (Figure 2E). Similarly, *HOXD13* was overexpressed in GBM compared to WHO Grade 3 glioma (confirmed by TCGA and CGGA), LGG (confirmed by TCGA, CGGA, Rembrandt, and qPCR), and normal brain tissue (confirmed by Rembrandt and qPCR). Furthermore, higher expression levels of all three genes were associated with worse overall survival across the TCGA, CGGA, and Rembrandt datasets (Figure 5, Table 3).

### *CD44* and *TNFSF14* distinguish between GBM and normal brain tissue and are associated with ME subtype

Using the Rembrandt dataset, we assessed how well the selected genes distinguish between GBM and normal brain tissue. Based on the ROC curve (Figure 6), *CD44* and *TNFSF14* demonstrate high sensitivity and specificity in differentiating GBM from normal brain tissue, with AUC values above 0.8. In contrast, *HOXD13* (AUC ═ 0.553) does not effectively distinguish between the two. Next, we examined the relationship between *CD44*, *TNFSF14*, and *HOXD13* and specific GBM subtypes (Figure 7). The results indicate that *CD44* and *TNFSF14* are more closely associated with the ME subtype in both the TCGA and Rembrandt classifications. In contrast, *HOXD13* does not show a clear association with any GBM subtype.

### *CD44* and *TNFSF14* are positively associated with Treg, resting NK cells and PD-L1 expression

We also analyzed the relationship between the expression of *CD44*, *TNFSF14*, and *HOXD13* with different immune cells (Table 4). *CD44* and *TNFSF14* showed a positive association with Tregs and resting NK cells, while they were negatively associated with activated NK cells. Additionally, CD44 was negatively associated with M2 macrophages and positively associated with resting memory CD4^+^ T cells.

**Table 4:** Association of *CD44*, *TNFSF14,* and *HOXD13* with immune cells in GBM

	***CD44***	***TNFSF14***	***HOXD13***
CD8+ T cells	−0.061	−0.079	0.003
CD4+ T cells naïve	−0.147	−0.088	−0.018
CD4+ T cells activated	0.056	0.059	0.072
CD4+ T cells memory resting	0.269	−0.032	−0.123
Treg	0.314	0.178	−0.043
Macrophage M0	−0.012	−0.017	0.144
Macrophage M1	−0.158	−0.08	−0.032
Macrophage M2	−0.214	−0.004	0.014
Myeloid DC activated	0.13	0.124	−0.058
Myeloid DC resting	0.041	−0.084	−0.09
NK cells activated	−0.277	−0.176	0.002
NK cells resting	0.291	0.188	−0.061

Data was obtained from online available platform TIMER 2.0, which provides information about association of immune infiltrates and genetic or clinical features [36]. Only results obtained from CIBERSORT were included. Statistically significant results (*P* < 0.05) are shown in orange (positive association) or blue (negative association). NK: Natural killer; TNFSF14: Tumor necrosis factor superfamily member 14; Treg: Regulatory T cell; CD44: Cluster of differentiation 44; GBM: Glioblastoma.

**Figure 8.**
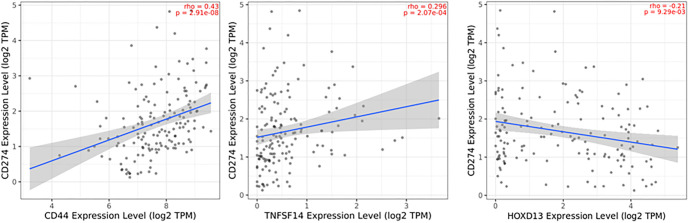
**The association of *CD44*, *TNFSF14,* and *HOXD13* with *CD274* (PD-L1) expression.** For CD44, the rho was 0.43, for TNFSF14 0.296, and for HOXD13 −0.21. CD44: Cluster of differentiation 44; TNFSF14: Tumor necrosis factor superfamily member 14; PD-L1: Programmed death-ligand 1.

Finally, we examined the association between the expression of these three genes and PD-L1 (Figure 8). The results indicate that CD44 has a moderate positive correlation with PD-L1 (*ρ* ═ 0.43), while TNFSF14 exhibits a weak positive correlation (*ρ* ═ 0.296). In contrast, *HOXD13* shows a weak negative association with PD-L1 expression (*ρ* ═ –0.21).

### *CD44*, *TNFSF14*, and *HOXD13* are related to both immunoinhibitory and immunostimulatory genes

**Figure 9.**
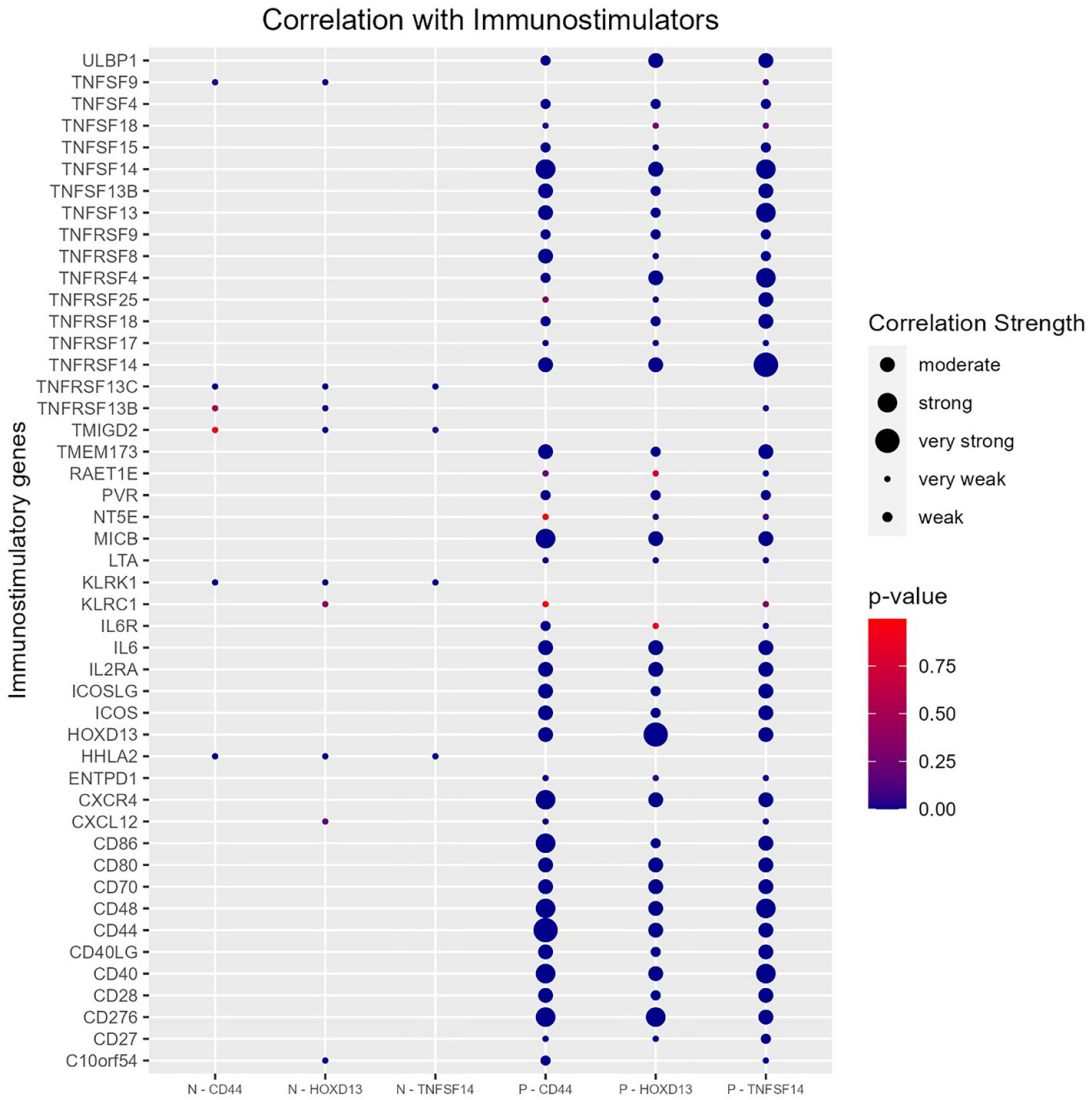
**Spearman correlation of *CD44*, *HOXD13,* and *TNFSF14* with immunostimulatory genes**. Genes were retrieved from TISIDB online database [37]. CD44: Cluster of differentiation 44; TNFSF14: Tumor necrosis factor superfamily member 14.

**Figure 10.**
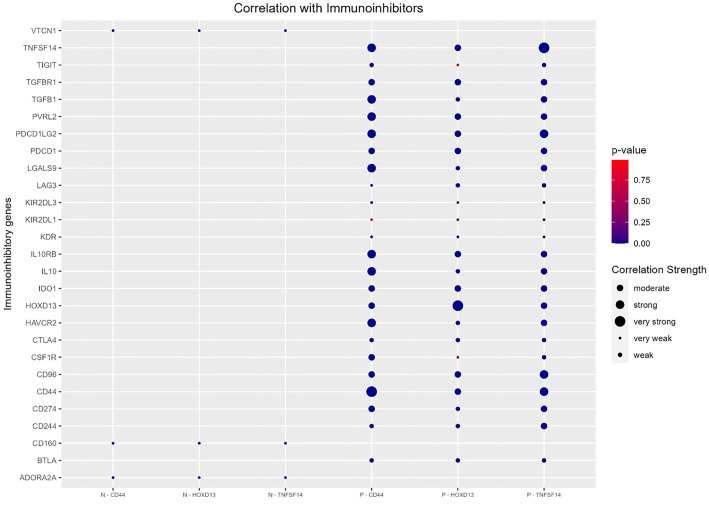
**Spearman correlation of *CD44*, *HOXD13,* and *TNFSF14* with immunostimulatory genes.** Genes were retrieved from TISIDB online database [37]. CD44: Cluster of differentiation 44; TNFSF14: Tumor necrosis factor superfamily member 14.

In the next step, we analyzed the relationship between *CD44*, *TNFSF14*, and *HOXD13* and both immunoinhibitory and immunostimulatory genes. These genes were selected from the TISIDB repository portal [37]. Our results indicate that all three genes exhibit a positive correlation with the majority of both immunostimulatory and immunoinhibitory genes. The strongest positive correlations among immunostimulatory genes were observed with *TNFRSF14*, *CD276*, *CD40*, and *CD48* (Figure 9). For immunoinhibitory genes, the strongest correlations were found with *PVRL2*, *IL10RB*, and *PDCD1LG2* (Figure 10).

**Figure 11.**
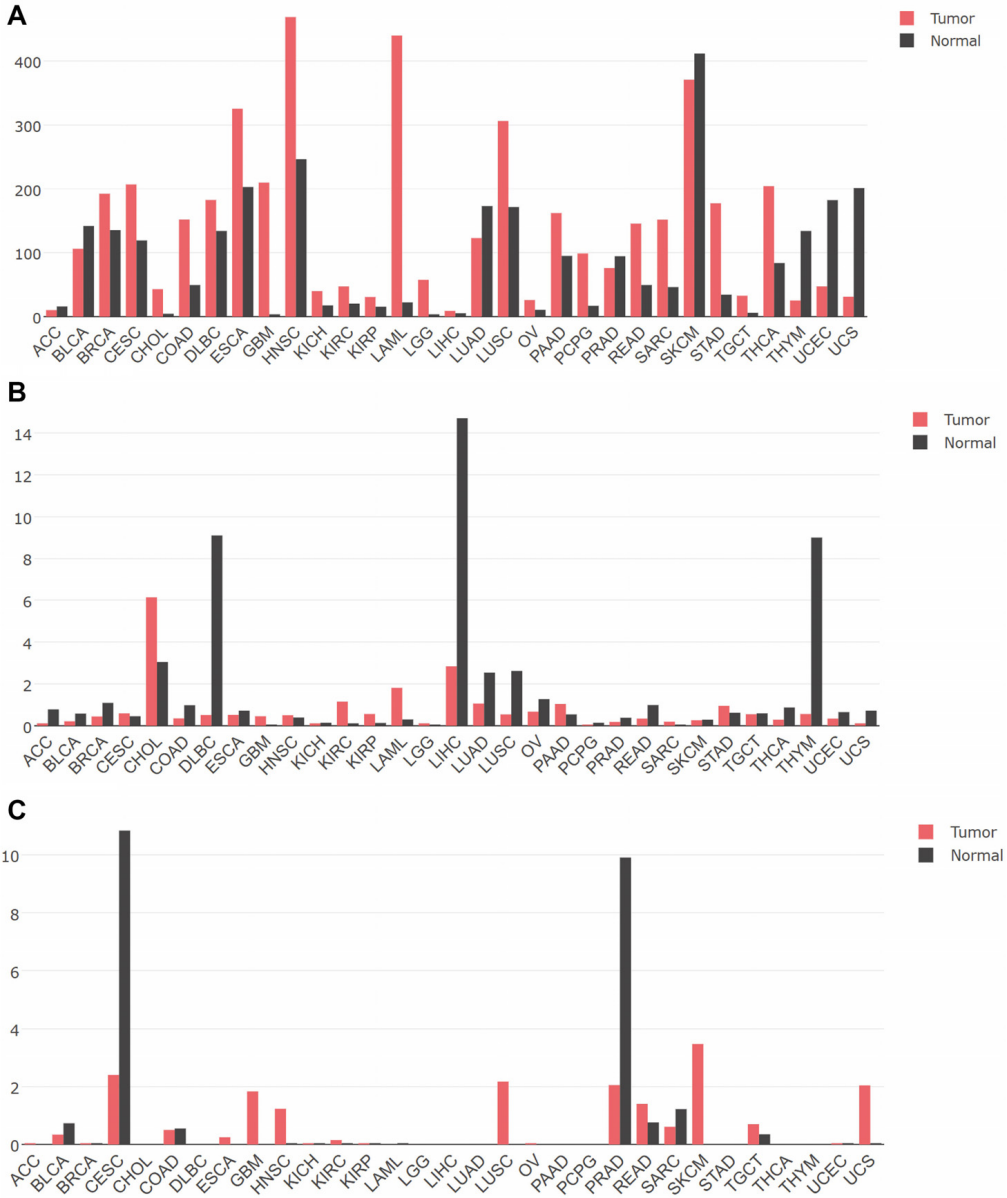
**Differential expression of (A) *CD44*, (B) *TNFSF14,* and (C) *HOXD13*.** Graphs were obtained from GEPIA online tool [38]. CD44: Cluster of differentiation 44; TNFSF14: Tumor necrosis factor superfamily member 14.

### Expression of *CD44*, *TNFSF14*, and *HOXD13* across different tumors and corresponding reference tissues

In the last step, we analyzed the gene expression patterns of *CD44*, *TNFSF14*, and *HOXD13* across various tumors compared to normal tissues using data from the GEPIA database [38] (Figure 11). For *CD44*, we observed a general trend of higher expression in tumor tissues across most cancer types studied (Figure 11A). However, notable exceptions included thymoma (THYM), uterine corpus endometrial carcinoma (UCEC), and uterine carcinosarcoma (UCS), where expression was higher in normal tissues than in tumors. In contrast, *TNFSF14* exhibited predominantly higher expression in normal tissues, with a general trend of downregulation in tumors (Figure 11B). Interestingly, there were exceptions in nine tumor types, where *TNFSF14* expression was elevated in tumor tissues compared to normal brain tissue, including GBM. Finally, *HOXD13* (Figure 11C) showed low or negligible expression in many tumors. However, it was significantly higher in normal tissues compared to tumors in cervical squamous cell carcinoma and endocervical adenocarcinoma (CESC) and prostate adenocarcinoma (PRAD). Conversely, in several cancers—including GBM, head and neck squamous cell carcinoma (HNSC), lung squamous cell carcinoma (LUSC), skin cutaneous melanoma (SKCM), and UCS—*HOXD13* was highly expressed in tumors but exhibited low or absent expression in the corresponding normal tissues.

## Discussion

GBM is one of the deadliest cancers, with a median survival rate of only 15 months [8]. Despite advancements in treatment, survival rates have not significantly improved, highlighting the urgent need for novel therapies. Immunotherapy, a rapidly developing field in cancer treatment, has shown limited success in GBM due to factors, such as high tumor heterogeneity, a lack of neoantigens, and an immunosuppressive environment [39–42].

However, recent studies suggest that immunotherapy may offer potential benefits in treating this aggressive cancer.

In our study, we aimed to explore the PD-L1-related gene network to identify potential new therapeutic targets. We constructed a PD-L1 network and analyzed data from three datasets (TCGA, CGGA, and Rembrandt). These three datasets were included because they comprise a large number of samples with detailed clinical reports, ensuring the robustness of our analysis.

Our analysis identified 15 genes overexpressed in GBM compared to WHO Grade 3 glioma, LGG, and normal brain tissue. These genes are also associated with shorter overall survival. Validating these findings with tissue samples from our biobank, we confirmed higher expression levels of three genes—*CD44*, *TNFSF14*, and *HOXD13*—in GBM.

CD44 is a transmembrane glycoprotein expressed in different isoforms and is involved in several key cancer processes, such as carcinogenesis, progression, and resistance to therapy [43]. It is a well-established biomarker of GBM stem cells and is frequently co-expressed with CD133, another important GBM stem cell biomarker [44]. Interestingly, CD44 is not exclusively GBM-specific; it is also expressed in astrocytes under normal physiological conditions [45]. Beyond GBM, CD44 has been implicated in aggressive behavior and tumorigenicity in multiple cancer types, such as breast, kidney, pancreatic, prostate, and gastrointestinal cancers [46].

TNFSF14, another protein overexpressed in GBM, as shown in our study, is expressed on activated T cells, NK cells, and immature DC cells. It exists both as a soluble protein and a type II transmembrane protein, interacting with two distinct receptors: HVEM and TLβR. The LIGHT-HVEM axis is particularly significant for its anti-tumor immune activity, as it facilitates the infiltration of CD8+ T cells into tumors, enhancing anti-cancer immune responses [47]. Notably, Long et al. [48] observed that TNFSF14 levels, in combination with immune checkpoint inhibitor genes, can predict survival prognosis for patients with GBM. Furthermore, TNFSF14 has been associated with disulfidptosis, a novel programmed cell death mechanism, and identified as one of the disulfidptosis-related immune checkpoints. Alongside genes, such as CD276, TNFRSF14, TNFSF4, CD40, and TNFRSF18, TNFSF14 has been incorporated into a comprehensive overall survival prediction model designed to evaluate sensitivity to immunotherapy [49].

Of the three genes discussed, the available data on HOXD13 is the most limited. HOXD13 is known to contribute to the pathogenesis of acute myeloid leukemia (AML) through the formation of a fusion protein with NUP98. Its expression patterns in cancer vary, with either overexpression or downregulation observed depending on the cancer type [50]. In GBM, however, research on HOXD13 remains sparse. A study by Zhang et al. [51] highlighted that HOXD13 is regulated by miR-7156-3p, a tumor suppressor, whereas HOXD13 itself exhibits oncogenic activity by promoting glioma stemness and tumor progression.

Even though it was found to be overexpressed in GBM, HOXD13 did not show the ability to differentiate between GBM and normal brain tissue and was not related to any specific GBM subtype. On the other hand, CD44 and TNFSF14 showed a distinct ability to differentiate between GBM and normal brain tissue and are associated with the ME subtype of GBM. CD44 has been previously linked to this subtype, as well as to increased invasion and proliferation in GBM [52–55]. TNFSF14 is also overexpressed in GBM and correlates with shorter overall survival and the ME subtype [48, 56, 57]. Our in-silico study, incorporating three independent large datasets, reaffirms these associations.

The association of both CD44 and TNFSF14 with the ME subtype is particularly significant, as this GBM subtype is characterized by heightened inflammation, poorer survival rates, and the worst prognosis compared to other subtypes [53, 58, 59]. The ME subtype is also linked to increased infiltration of TAMs, which further worsens its aggressive nature [60]. Notably, in cases of recurrence, the proneural subtype frequently transitions to the ME subtype, leading to a more aggressive tumor phenotype and worse clinical outcomes [61].

CD44 and TNFSF14 are both associated with resting NK cells. NK cells, when in an active state, exhibit high antitumor and antimetastatic activity [62]. However, tumors can evade NK cell surveillance when these cells are not in an active state but instead exist as tumor-exposed NK cells, which have a gene expression profile similar to that of resting NK cells. In GBM, the positive association between CD44 and TNFSF14 expression and resting NK cells suggests that these genes may be involved in immune evasion and suppression. Additionally, CD44 and TNFSF14 were found to be positively associated with Tregs, which are known to suppress the immune response and promote tumor growth. Treg cells are major contributors to the immune-suppressive environment [63–65].

Hu et al. developed a prognostic model based on natural killer T (NKT) cells, incorporating single-cell RNA sequencing (scRNA-seq) data from the GEO database, as well as datasets from TCGA and CGGA. This model includes two key NKT markers, CD44 and TNFSF14 [66]. Xiao et al. further highlighted the role of CD44 in glioma immunity, demonstrating that CD44+ tumor cells predominantly exist in a ME-1-like cellular state. Additionally, CD44+ T cells exhibit high expression levels of PD-1 and PD-L1, emphasizing their role in immune regulation [67]. Notably, CD44 expression is positively correlated with PD-L1 levels in GBM patients, a relationship also observed in triple-negative breast cancer (TNBC) patients. CD44 has even been proposed as a potential target for modulating PD-L1 function, as it triggers transcriptional activation of PD-L1 through its intracytoplasmic domain [68]. In TNBC, tumors with high PD-L1 expression are associated with enhanced immune and cancer stemness pathways, further elucidating their connection to CD44.

Besides CD44, a weaker but positive correlation between TNFSF14 and PD-L1 expression has also been observed. Similarly, Yang et al. reported a significant association between TNFSF14 and immune checkpoints, such as the PD-1/PD-L1 pathway, TIM-3, and B7-H3 [56].

To fully understand these findings, functional studies are essential. Co-culture experiments of GBM cells with immune cells and gene knockout, combined with advanced techniques like single-cell sequencing, could provide deeper insights into the mechanisms underlying these correlations and their potential for therapeutic exploitation.

## Conclusion

In this study, by integrating three independent datasets, we revealed insights into the molecular behavior of GBM, particularly highlighting the roles of *CD44*, *TNFSF14*, and *HOXD13*. *CD44* and *TNFSF14* emerged as promising therapeutic targets in GBM due to their significant overexpression compared to normal brain tissue and their association with the ME subtype, which is linked to heightened inflammation, poorer prognosis, and immune suppression. Both genes also correlate with immune evasion mechanisms involving resting NK cells and Tregs, underscoring their role in shaping the TME. Additionally, CD44 and TNFSF14 are closely associated with PD-L1 expression, suggesting a potential role in regulating immune checkpoint pathways. To confirm the roles of CD44 and TNFSF14 as potential targets, functional studies—such as co-culture experiments of GBM cells with immune cells, gene knockout models, and advanced techniques like single-cell sequencing—are required. These studies could provide deeper mechanistic insights and support the development of novel, more effective therapeutic strategies for GBM.

## Supplemental data

**Figure S1.**
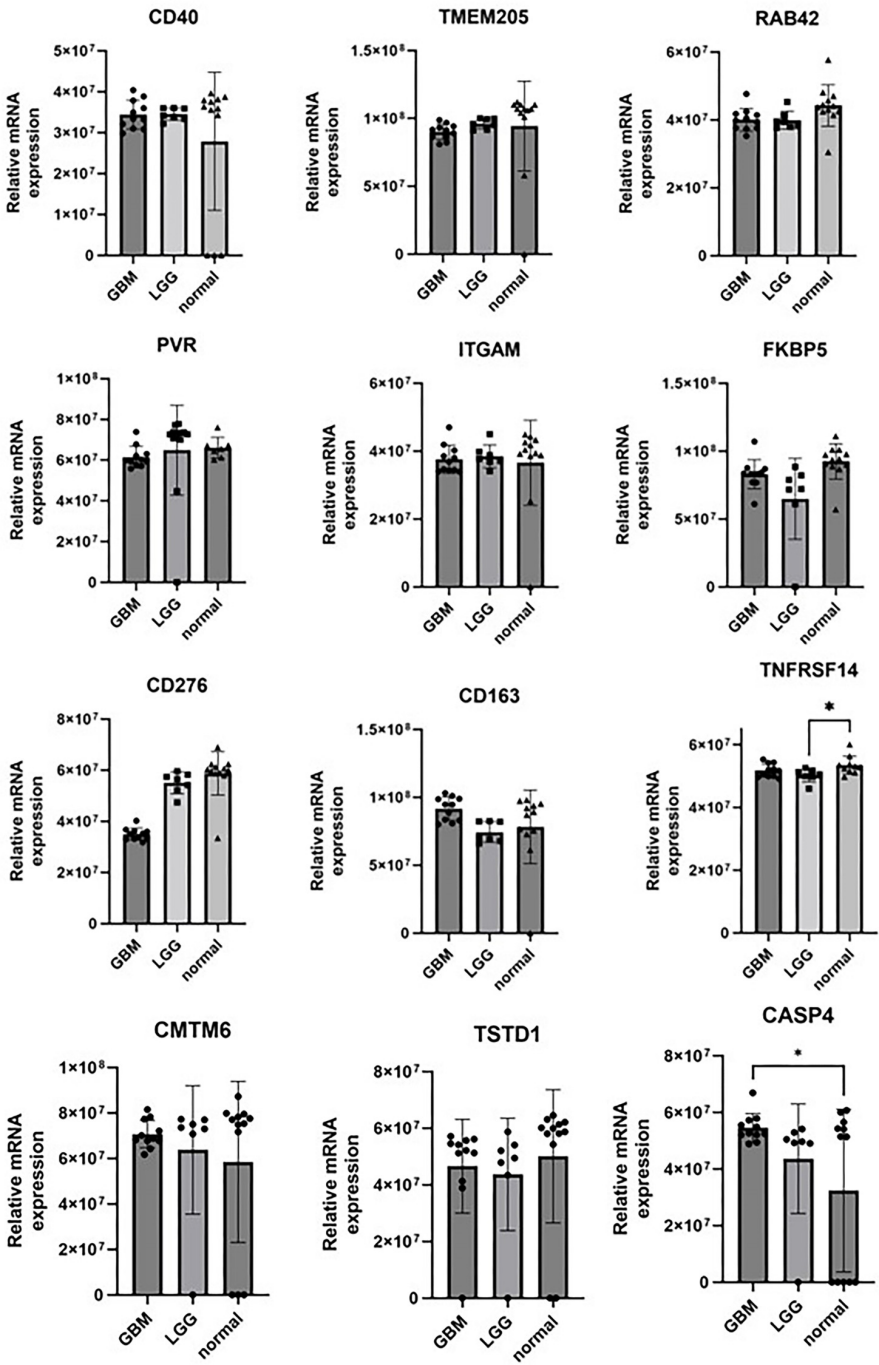
**Gene expression in human tissue samples.** Results are presented as mean +/− SD. *P < 0.05, **P < 0.01, ***P < 0.001, ***P < 0.0001.

## Data Availability

Data is available at the corresponding author upon request.
